# Anionic lanthanide complexes with 3-methyl-1-phenyl-4-formylpyrazole-5-one and hydroxonium as counter ion

**DOI:** 10.1016/j.ica.2013.03.044

**Published:** 2013-06-01

**Authors:** Victor F. Shul’gin, Oleg V. Konnik, Susana V. Abkhairova, Alexey N. Gusev, Svetlana B. Meshkova, Anna V. Kiriyak, Eduard B. Rusanov, Miki Hasegawa, Wolfgang Linert

**Affiliations:** a Taurida National V.I. Vernadsky University, Simferopol 95007, Ukraine; b A.V. Bogatsky Physico-Chemical Institute of the National Academy of Sciences of Ukraine, Odessa 65080, Ukraine; c Institute of Organic Chemistry, Academy of Sciences of Ukraine, Kiev 02094, Ukraine; d Department of Chemistry and Biological Science, College of Science and Technology, Aoyama Gakuin University, Kanagawa 252-5258, Japan; e Institute of Applied Synthetic Chemistry, Vienna University of Technology, Getreidemarkt 9/163, A-1060 Vienna, Austria

**Keywords:** 4-Formyl-pyrazol-5-one, Lanthanide complexes, Crystal structure, Luminescence

## Abstract

A series of [H_3_O]^+^[LnL_4_]^−^·*n*H_2_O complexes (*n* = 1–3, Ln = Nd, (**1**), Sm (**2**), Eu (**3**), Tb (**4**); HL = 3-methyl-1-phenyl-4-formylpyrazole-5-one) were synthesized and characterized. The structures of the Sm^III^ and Eu^III^ complexes were investigated by X-ray diffraction. The isostructutal crystalls **2** and **3** consist the tetrakis [LnL_4_]^−^ anions which are linked by H-bonding with the hydroxonium counter-ion and water molecules. The lanthanide ion is situated in the center of distorted tetragonal antiprism formed by eight oxygen atoms of 4-formyl-5-hydroxypyrazolonate anions. The Tb^III^ and Sm^III^ complexes show strong luminescence in solid state, whereas the Eu^III^ and Nd^III^ complexes show low luminescence activity.

## Highlights

• Anionic Ln(III) complexes with 3-methyl-1-phenyl-4-formylpyrazole-5-one.
• X-ray crystallographic analysis of Ln(III) complexes.
• Ion luminescence of Ln(III) in solid state.
• The rare case of stronger Sm(III) luminescence compared with an analogs Eu(III) compound.

## Graphical abstract

New lanthanide (Ln) coordination compounds of the composition [H_3_O][LnL_4_]·*n*H_2_O (*n* = 1–3, Ln = Nd, Sm, Eu, Tb; HL – 3-methyl-1-phenyl-4-formylpyrazol-5-one) with hydroxonium cation as counter were synthesized and investigated by various means, showing the rare case of stronger Sm(III) luminescence compared with an analogues Eu(III) compound.

**Figure:**
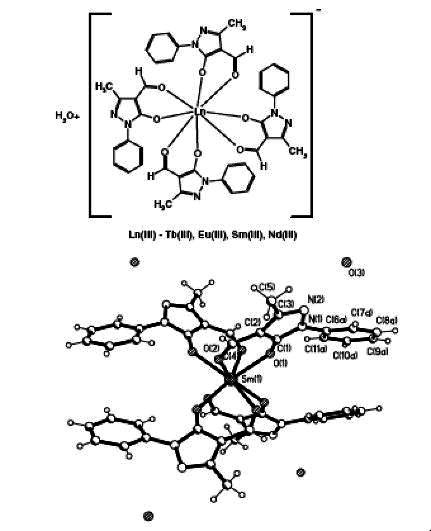

## 1 Introduction

Investigation of coordination compounds of lanthanide ions has been attracted significant attention, that are focuses on several potential applications of its luminescence: application in the lighting industry for the engineering of lamps, ability to provide electroluminescent material for organic light emitting diodes (OLEDs) and optical fibers for telecommunications, a capacity to yield functional complexes for biological assays and medical imaging purposes [1], [2], [3].

Among lanthanides coordination compounds, which exhibit strong luminescence in visible range, the Eu^III^ and Tb^III^ complexes are most studied. Characteristic red and green intensive luminescence, respectively, due to structure of their energy levels are typical for them. However, there are also Ln compounds that emit light in other spectral regions such as near infrared (Nd^III^, Yb^III^ and Er^III^), visible (orange for Sm^III^, yellow for Dy^III^, blue for Tm^III^) and near-UV (Ce^III^ and Gd^III^) range.

Some types of organic ligands are convenient for the molecular design of luminescent lanthanides complexes, among them are β-diketones, carboxylic acids and acylpyrazolones [4]. 4-Acyl-5-pyrazolones are an interesting class of β-diketones, containing a pyrazole fused to a chelating arm. The results of acylpyrazolones complexes study are summarizes in some reviews for example [5]. However, coordination compounds of some ligands of this type have not investigated till now. Recently we described the peculiarities of molecular structure and luminescent properties of lanthanides complexes with 3-methyl-1-phenyl-4-formylpyrazole-5-one (HL) of the general formula LnL_3_·*n*Solv (Solv = H_2_O, EtOH, Ln = La, Nd, Sm, Eu, Gd, Tb, Dy, Yb) [6].

**Figure:**
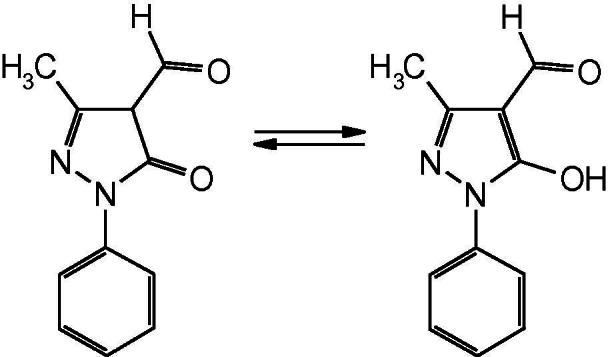

It was found that solvent molecules in the inner coordination sphere are able to effectively quench luminescence of lanthanides due to the transfer of excitation energy to their oscillating levels. The replacement of ОН-oscillators in the coordination sphere via formation of anionic complexes [Na(H_2_O)][LnL_4_] and [NBu_4_][LnL_4_] yields to a considerable increasing of luminescence intensity [7]. The high stability of the [LnL_4_]^−^ anions allowed us to synthesize the complexes of the composition [H_3_O]^+^[LnL_4_]^−^ (Ln = Nd, Sm, Eu, Tb), which belong to the rare type of lanthanides coordination compounds. The first structurally characterized β-diketonate acid stabilized by hydrogen bonding are described only 10 years ago [8].

## 2 Results and discussion

### 2.1 Synthesis and characterization

3-Methyl-1-phenyl-4-formylpyrazole-5-one is a structural analog of β-diketones and easily forms coordination compounds in deprotonated enol form. The reaction of sodium salt of 3-methyl-1-phenyl-4-formylpyrazole-5-one and lanthanides salts in molar ratio 4:1 leads to the complex salts [Na(H_2_O)]^+^[LnL_4_]^−^ [7]. The synthesis of complexes [H_3_O]^+^[LnL_4_]^−^ with hydroxonium as a counter-ion with high yields was realized by molar ratio of HL:NaOH:Ln^III^ as 4:3:1.

The presence of the ligand in a coordinating sphere in a deprotonated form is confirmed by the IR-spectroscopy data. Thus, the formation of complexes **1**–**4** result in disappearance of bands with maxima absorption in range of 1690 and 1668 cm^−1^, corresponding the stretching oscillation of aldehyde group of different molecular forms of 3-methyl-1-phenyl-4-formylpyrazole-5-one. Moreover considerable intensity of absorption band with a maximum at 1633–1635 cm^−1^ caused by the stretching oscillation of double carbon–nitrogen bond increases and new intensive duplicate line with a maximum of absorption in range of 1364–1367 cm^−1^, that is absent in the spectrum of free acylpyrazolone, appeared. These lines are usually attributed to the stretching oscillation of exo- and endo-cyclic carbonyl groups of 4-acyl-pyrazole-5-one [5].

The stretching oscillation of ОН-bonds of the water molecules show up in IR-spectra of compounds **1**–**4** as wide poorly resolved line with maximum of absorption in range 3342–3429 and 3229–3266 cm^−1^.

According to the thermal analysis data it was found that the dehydration of **1**–**4** complexes take place in a range of 100–200 °C. It is accompanied with a strong endothermic effect with a minimum on the DTA curve at 170 °С. Besides 210–260 °C on the curve of TGA there is another endoeffect which is not accompanied by changing of mass and, presumably, it is caused by the process of sample melting. Increasing of temperature up to 280–300 °C results in thermo-oxidative destruction of ligand. The process is accompanied by series of insignificant thermal effects and passes onto the process of burning down of organic residuum with a powerful maximum on the curve of DTA at 540–600 °С. The thermal decomposition process of samples accomplishes at 700–720 °С.

### 2.2 Crystal structure descriptions

**Fig. 1:**
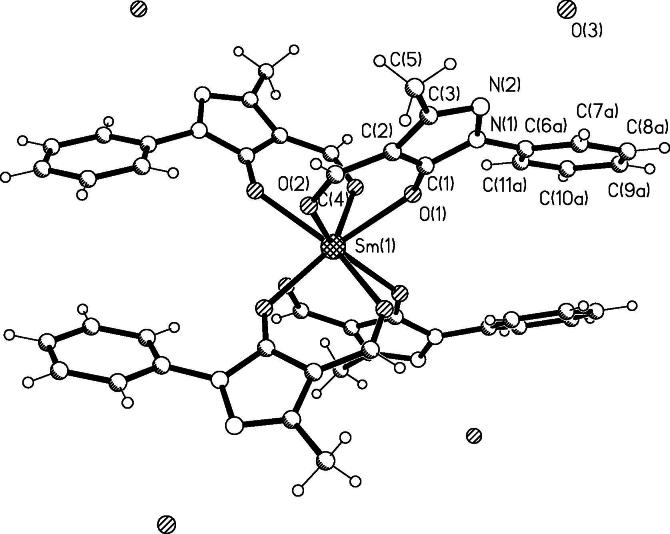
Structure of complex [H_3_O]^+^[SmL_4_]^−^·H_2_O (compound **2**,**а**). Four positions of the disorientated water molecules are shown. Selected bond lengths (Å) and bond angles (°): Sm(1)–O(1) 2.378(5), Sm(1)–O(2) 2.464(5), N(1)–N(2) 1.422(8), O(1)–C(1) 1.271(8), O(2)–C(4) 1.234(8), C(1)–C(2) 1.414(8), C(2)–C(4) 1.416(9); O(1)Sm(1)O(1) 80.6(2), O(1)Sm(1)O(1) 111.6(2), O(1)Sm(1)O(1) 142.6(2), O(1)Sm(1)O(2) 75.01(15), O(1)Sm(1)O(2) 144.22(17), O(1)Sm(1)O(2) 72.03(18), O(1)Sm(1)O(2) 75.86(16), O(2)Sm(1)O(2) 137.5(2), O(2)Sm(1)O(2) 119.4(2), O(2)Sm(1)O(2) 76.8(3). Selected bond lengths (Å) for [H_3_O]^+^[EuL_4_]^−^·H_2_O (compound **3**): Eu(1)–O(1) 2.381(4), Eu(1)–O(2) 2.445(5), N(1)–N(2) 1.422(7), O(1)–C(1) 1.254(7), O(2)–C(4) 1.242(7), C(1)–C(2) 1.421(8), C(2)–C(4) 1.417(8).

X-ray diffraction analysis of crystals than were obtained by the crystallization of complex **2** and **3** from methanol, was performed. It was found that the complex **2** loses part of water and in result [Н_3_О][SmL_4_]·H_2_O (compound **2**,**a**) formed. In the crystal structures of **2**,**a** and isostructural **3**, the Ln ion is eight-coordinate (Fig. 1). It is situated in the center of distorted tetragonal antiprism formed by eight oxygen atoms of ligands.

Chelate cycles are practically planare, deviations of Sm^III^ and Eu^III^ cation from a chelate planes are 0.014 and 0.006 Å, respectively. The chelating ligands are structurally equivalent. Bonds lengths of Samarium cation and endo- and exocyclic oxygen atoms differs considerably (2.377(5) and 2.464(5) Å, respectively), that is caused by asymmetric coordination of ligand. Bond lengths of Europium cation and oxygen atoms has similar values (2.382(3) and 2.464(5) Å, respectively). At the same time comparison of lengths of carbon–oxygen bonds (C(1)–O(1) 1.270(8), 1.253(7) Å, and C(4)–O(2) 1.234(8), 1.242(8) Å for Sm^III^ and Eu^III^ complexes, respectively) testifies to the high degree of double bonds delocalization, that are typical for β-diketonates. Bonds lengths and valency angles in the organic ligands are near to the ordinary volumes [9].

Two molecules of water are located in an external sphere and are diffused up on four positions with equal population. In all positions water molecules are connected by H-bonds with the nitrogen atoms of pyrazole heterocycles (O(3)⋯N(2), 2.706 Å). Strictly localization of hydrogen cation was not successful. Most probably, that one are dislocated between the molecules of water in all four positions. Being involved in H-bonding hydroxonium ion and water molecule stabilized the crystal structures.

### 2.3 Luminescent properties

It is well known that the luminescence of trivalent lanthanide ions is realized as a result of the energy transfer from the excited organic moiety of the complex molecule [1]. The formation of the stable complexes compound is a necessary but insufficient condition; it is important that the energy of the lower triplet level of the ligand should be equal or higher than the energy of the excited level of the lanthanide ion. Experimentally found values of singlet (*E*_S1_) and triplet (*E*_T1_) energy levels of 3-methyl-4-formyl-1-phenylpyrazole-5-one are 24 150 and 20 700 cm^−1^, respectively. High value of *E*_T1_ of ligand makes possible energy transfer both on the highly located irradiative levels of Terbium^III^ (*E*(^5^D_4_) = 20 500 cm^−1^) and Samarium^III^ (*E*(^4^G_5/2_) = 17 920 cm^−1^) and the below located level of Europium^III^ (*E*(^5^D_0_) = 17 250 cm^−1^) and Neodymium^III^ (*E*(^4^F_3/2_) = 11 500 cm^−1^).

**Fig. 2:**
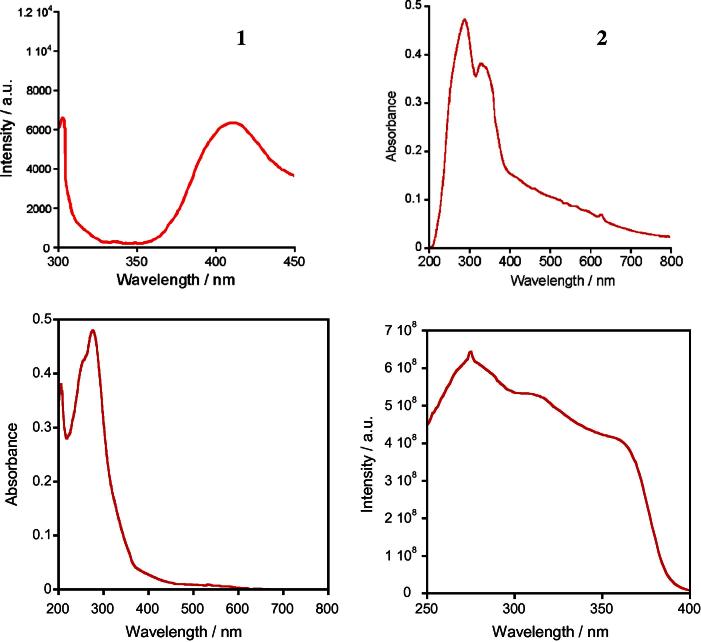
Fluorescence spectrum (*λ*_exc_ = 270 nm) of ligand – 1, absorption spectra of complexes [H_3_O][TbL_4_]·H_2_O – 2 and [H_3_O][EuL_4_]·H_2_O – 3 and excitation spectrum of [H_3_O][TbL_4_]·H_2_O – 4.

The intensive band of absorption with maximum at 300 nm was detected in the electronic spectra of complexes **1**–**4**. The excitation spectra a quite similar for all complexes and contain a broad band in the region 250–350 nm. Fig. 2 shows the fluorescence spectrum of ligand and absorption spectra of Tb^III^ and Eu^III^ complexes and excitation spectrum of Tb^III^ complex as example.

**Fig. 3:**
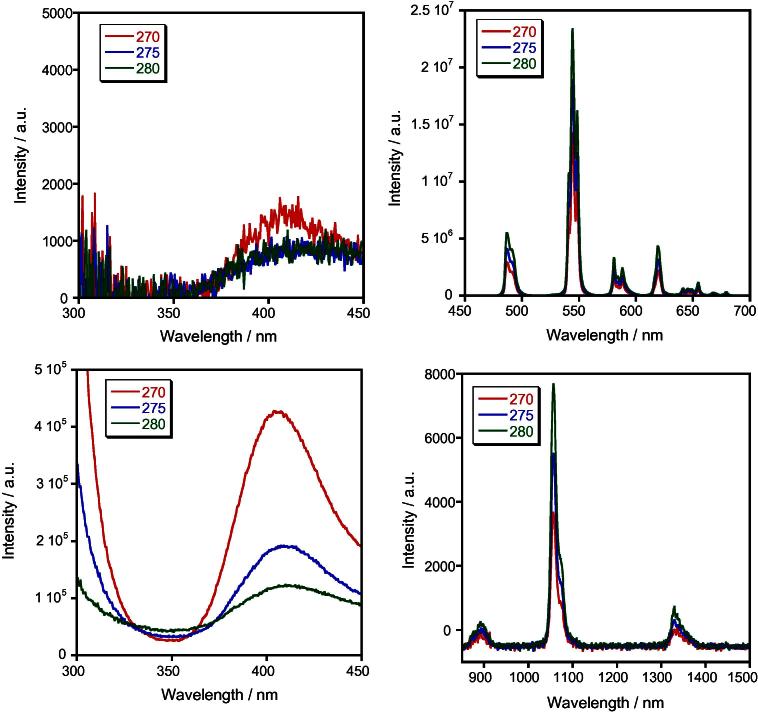
Luminescence spectra of solid complexes: [H_3_O][TbL_4_]·H_2_O (top) and [H_3_O][NdL_4_]·2H_2_O (bottom) at different excitation energies.

According to the luminescence spectra the excitation energy transfer from ligand to ion Ln^III^ occurs more effectively in case of Tb^III^ and Sm^III^, less – to Eu^III^ and least of all – to Nd^III^ (Fig. 3). Values of the luminescence intensity and quantum yields of complexes are presented in the Table 1.

**Table 1:** Characteristics of luminescence of lanthanides complexes with 3-methyl-1-phenyl-4-formyl-pyrazole-5-one (*λ*_exc_ = 365 nm).

Complex	f–f transition	*λ*_max_ (nm)	*I*_lum_, a.u.^a^	Quantum yield of luminescence (%)
[Н_3_О][NdL_4_]·2H_2_O	^4^F_3/2_→^4^I_11/2_	1060	7.6	–
[H_3_O][SmL_4_]·3H_2_O	^4^G_5/2_→^6^Н_5/2_	560	340	0.75
	^4^G_5/2_→^6^Н_7/2_	605	960	
	^4^G_5/2_→^6^Н_9/2_	645	1500	
[H_3_O][EuL_4_]·H_2_O	^5^D_0_→^7^F_2_	611	36.5	0.08
	^5^D_0_→^7^F_4_	700	5.0	
[H_3_O][TbL_4_]·H_2_O	^5^D_4_→^7^F_6_	488	6500	27.9
	^5^D_4_→^7^F_5_	546	21 200	

a Value *I*_lum_ are corrected to the identical conditions of experiment.

The luminescence spectrum of Neodymium complex contains three bands: the first with maximum at 896 nm, the second – at 1060 nm, and the third – at 1320 nm, corresponding to the transitions from the irradiative level ^4^F_3/2_ on sublevels of the basic level ^4^I_9/2_, ^4^I_11/2_ and ^4^I_15/2_, respectively, that are also observed in the spectra of complexes of Nd^III^ with β-diketones and other reagents [2].

In the spectrum of Sm^III^ complex in range 550–660 nm three bands corresponding to the transitions from the irradiative level ^4^G_5/2_ on the sublevels of basic level ^6^Н_5/2,__7/2,__9/2_ (*λ*_max_ = 560, 600, 645 nm respectively) are observed. The transition ^4^G_5/2_→^6^H_9/2_ (electric-dipole transition) at around 645 nm the most dominated. Tb^III^ complex displays a typical emission spectrum with emission peaks centered at 489, 546, 590, 621 and 650 nm corresponding to the ^5^D_4_→^7^F_J_ (*J* = 6, 5, 4, 3, 2) transitions respectively. The ^5^D_4_→^7^F_6_ band is the most dominate one.

**Fig. 4:**
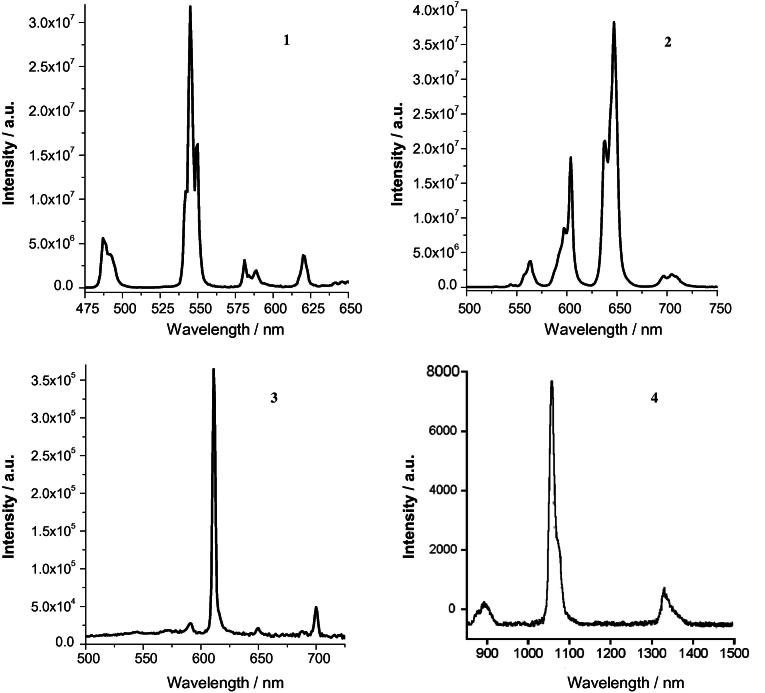
Luminescence spectra of solid complexes: [H_3_O][TbL_4_]·H_2_O (*λ*_exc_ = 345 nm) – 1, [H_3_O][SmL_4_]·3H_2_O (*λ*_exc_ = 345 nm) – 2, [H_3_O][EuL_4_]·H_2_O (*λ*_exc_ = 371 nm) – 3, [H_3_O][NdL_4_]·2H_2_O (*λ*_exc_ = 280 nm) – 4.

In all cases the strong Stark’s spliting of bands corresponding to the hypersensitive transitions (HST) is observed: ^4^F_3/2_→^4^I_9/2_ of Nd (*λ*_max_ = 872, 896 nm), ^4^G_5/2_→^6^Н_5/2_ of Sm (*λ* = 640, 645 nm) and ^5^D_4_→^7^F_5_ of Tb (*λ* = 542, 546, 550 nm) due to of distortion of the coordinating polyhedron geometry (Fig. 4).

As it was expected the IR-luminescence of Nd^III^ is far weaker in comparison with the complexes of Sm^III^ and Tb^III^ radiating in visible range of spectrum. Thus, the high quantum yield of luminescence of Tb^III^ (28%) is near to previously observed quantum yield for the complex with 6-[(benzylamino)carbonyl]-2-pyridinecarboxy acid (34%), for example [10]. The discovered high-efficiency luminescence of Tb^III^ and high quantum yield of luminescence its anionic complex with 3-methyl-4-formyl-1-phenylpyrazole-5-one provides a basis to suppose that this compound can be used as irradiative material in OLEDs.

The Eu^III^ complex is isomorphic to the complex of Sm^III^ but does not show the expected strong red luminescence. The luminescence spectra of Eu^III^ complex display typical pattern of europium ^5^D_0_→^7^F_J_ transitions (*J* = 0–4) emissive transitions with the most prominent band at 610–612 nm assigned to the ^5^D_0_→^7^F_2_ dominating transition. Usually Eu^III^ complexes show a more intense luminescence, as in Sm^III^ complexes is dissipation of excitation energy in the high-lying ground state sublevels. According to the literature data for an efficient energy transfer to the emitting level of the Eu^III^ the difference between the triplet level of the organic ligand and the resonance level of the lanthanide should be in the range of 2500–3500 cm^−1^ [11]. In the europium complexes with the 3-methyl-4-formyl-1-phenylpyrazole-5-one energy gap between the triplet level of the ligand and the emitting level of the central atom (3450 cm^−1^) is at the upper limit of the optimal range making the transfer of excitation energy inefficient, and molecular phosphorescence of ligand is observed instead of the expected lanthanide ion luminescence. At the same time, the energy gap between the triplet level of the ligand and the resonance level of the Sm^III^ cation (2900 cm^−1^) falls in the middle of the optimal range, making an efficient energy transfer to the resonance level, and the luminescence intensity is higher than that of the europium complex.

It should be noted that the authors of work [12] also discovered that the quantum yield of luminescence of Sm^III^ complex with 1-phenyl-3-methyl-4-phenylacetylpyrazole-5-one is six times more than for analogical Eu^III^ complex, although its luminescence in similar europium complexes is usually 10–100 times higher [1], [2]. A same situation was described for the anionic complexes of Eu^III^ and Sm^III^ with the ligands obtained by condensation of salicylic acid hydrazide and 1-phenyl-3-methyl-4-propionylpyrazole-5-one [13].

## 3 Conclusion and perspectives

Summarizing, we have successfully synthesized new anionic lanthanideIII complexes based on 3-methyl-1-phenyl-4-formylpyrazol-5-one (HL) and the hydroxonium cation as a counter ion. High efficiency luminescence and high luminescence quantum yield of the complex [Н_3_О][TbL_4_]·H_2_O uniquely exhibits that this compound can be used as irradiative material in OLEDs.

## 4 Experimental

### 4.1 Materials and methods

Commercially available solvents and lanthanides salts were used without further purification. 3-Methyl-1-phenyl-4-formylpyrazole-5-one was prepared according to the literature procedure [14].

IR spectra were recorded for KBr pellets in the range of 4000–400 cm^−1^ on a Nicolet Nexus 470 FT IR-spectrometer. Thermal analysis experiments were performed on a Paulik–Paulik–Erdey Q-derivatograph under static air atmosphere and calcinated alumina as standard. Luminescence and excitation spectra of solid samples were recorded on Horiba Jobin–Yvon Fluorolog FL3-22 spectrometer equipped with a 450 W Xe-lamp and LOMO SDL-1 spectrometer equipped with a FEU-79 (visible) and FEU-62 (IR range) photomultiplier. The energy of the ligand triplet state (*E*_T1_) was determined on the basis of a low temperature (77 K) phosphorescence spectrum of Gd^III^ complex in DMSO solution. Quantum yield for solid samples was determined via a direct method using the home-modified integrating sphere [15]. Estimated errors of the values of quantum yields are 10%.

### 4.2 Synthesis of [H_3_O][Ln(L)_4_]·*n*H_2_O (**1**–**4**), general procedure

3-Methyl-1-phenyl-4-formylpyrazole-5-one (0.808 g, 4 mmol) was added to a solution of 0.12 g (3 mmol) sodium hydroxide in 25 ml 96% ethanol. The mixture was stirred at ambient temperature until complete dissolution of the precipitate (20–30 min). Then a solution of 1 mmol lanthanide chloride in 10 ml of ethanol was added and again stirred for half an hour. The resulting mixture was filtered off and left for the crystallization. The obtained crystals residue, which appeared after 24 h, was separated by filtration, washed on a filter with a small amount of ethanol and then dried in the air. The yields were 50–80%. Elemental analyses data and characteristic IR-absorption bands of the synthesized compounds are given below:

[Н_3_О][NdL_4_]·2H_2_O (**1**). *Anal.* Calc. for C_44_H_43_N_8_O_11_Nd: С, 52.63; Н, 4.32; N, 11.16. Found: С, 53.03; Н, 4.65; N, 10.64%. IR (KBr, сm^−1^): *ν* = 3365, 3231 *ν*(OH); 1633, 1364 *ν*(СО).

[Н_3_О][SmL_4_]·3H_2_O (**2**). *Anal.* Calc. for C_44_H_45_N_8_O_12_Sm: С, 51.40; Н, 4.41; N, 10.89. Found: С, 51.70; Н, 4.51; N, 10.52%. IR (KBr, сm^−1^): *ν* = 3429, 3266 *ν*(OH); 1635, 1366 *ν*(СО).

[Н_3_О][EuL_4_]·H_2_O (**3**). *Anal.* Calc. for C_44_H_41_N_8_O_10_Eu: С, 53.18; Н, 4.16; N, 11.27. Found: С, 53.04; Н, 4.57; N, 10.69%. IR (KBr, сm^−1^): *ν* = 3377, 3229 *ν*(OH); 1634, 1366 *ν*(СО).

[Н_3_О][TbL_4_]·H_2_O (**4**). *Anal.* Calc. for C_44_H_41_N_8_O_10_Tb: С, 52.81; Н, 4.13; N, 11.20. Found: С, 52.65; Н, 4.24; N, 10.84%. IR (KBr, сm^−1^): *ν* = 3342, 3240 *ν*(OH); 1635, 1367 *ν*(СО).

### 4.3 X-ray crystallography study

Single crystal structures determination of 2,a and 3 by X-ray diffraction was performed on a Bruker Apex-II CCD diffractometer (Мо Kα radiation, graphite monochromator, *λ* = 0.71073 Å) at 296 K following standard procedure [16]. The structures were solved by direct methods and were refined by the full-matrix least square fits with anisotropic thermal parameters for all non-hydrogen atoms. The hydrogen atoms of the carbon-containing ligands were positioned geometrically and refined using the ‘riding’ model. All calculations were carried out using the shelxtl program package [17]. The crystallographic parameters are given in Table 2.

**Table 2:** Crystal data and structure refinement statistics for **2**,**a** and **3**.

Parameter	**2**,**a**	**3**
Formula	C_44_H_41_N_8_O_10_Sm	C_44_H_41_N_8_O_10_Eu
Formula weight (g mol^−1^)	992.20	993.81
Crystal dimensions (mm)	0.08 × 0.09 × 0.17	0.10 × 0.10 × 0.32
*T* (K)	296	296
Crystal system	tetragonal	tetragonal
Space group	$I\overset{¯}{4}c2$	$I\overset{¯}{4}c2$
*Unit cell parameters*		
*а* (Å)	16.2701(4)	16.3271(3)
*b* (Å)	16.2701(4)	16.3271(3)
*c* (Å)	16.1315(6)	16.1084(8)
*V* (Å^3^)	4270.3(2)	4294.1(2)
*Z*	4	4
*D*_calc_ (g сm^−3^)	1.543	1.537
*μ* (mm^−1^)	1.444	1.529
*F*(0 0 0)	2012	2016
*θ*_max_ (°)	28.14	28.30
Index ranges	−21 ⩽ *h* ⩽ 21 −21 ⩽ *k* ⩽ 20 −21 ⩽ *l* ⩽ 21	−21 ⩽ *h* ⩽ 21 −20 ⩽ *k* ⩽ 21 −21 ⩽ *l* ⩽ 21
Reflections measured/reflections independent/reflections with *I* > 2(*σ*)	26 102/2616/1822	33 086/2666/1883
*R*_int_	0.1090	0.0883
*R* (all data)	0.0857	0.0783
*R*_1_ (*I* > 2*σ*(*I*))	0.0488	0.0452
*wR*_2_	0.1147	0.0992
Goodness-of-fit (GOF) on *F*^2^	1.078	1.024
Residual electron density (max/min) (е/Å^3^)	1.881/−1.242	1.671/−1.104
